# Supplementary material for: Artificial intelligence-based point-of-care lung ultrasound for screening COVID-19 pneumoniae: Comparison with CT scans
Source: PLoS One. 2023 Mar 16;18(3):e0281127. doi: 10.1371/journal.pone.0281127 (PMC10019704; doi:10.1371/journal.pone.0281127)
Supplement: Attachment — Submitted filename: Reponse to Reviewers AI_LungPOCUS2.docx [file pone.0281127.s001.docx]

# Dear Editors and Reviewers,

We appreciate your valuable comments and suggestions. Based on these, we revised our manuscript as explained in the response to the reviewers attached below. **The reviewers’ comments are noted in Bold** followed by our response. The major changes include the newly added 95% confidence intervals for each metric and the PPV and NPV calculated assuming the prior probabilities. We believe these changes made the manuscript more scientific and robust. We will be glad if the revised manuscript is worth publishing in your journal.

Best regards,

# Journal Requirements:

**When submitting your revision, we need you to address these additional requirements.**

## 1.

**Please ensure that your manuscript meets PLOS ONE's style requirements, including those for file naming. The PLOS ONE style templates can be found at**

**https://journals.plos.org/plosone/s/file?id=wjVg/PLOSOne_formatting_sample_main_body.pdf and https://journals.plos.org/plosone/s/file?id=ba62/PLOSOne_formatting_sample_title_authors_affiliations.pdf.**

Response: We read it and confirmed that the style of our manuscript follows it.

## 2.

**Thank you for stating in your Funding Statement:**

**"This work was partially supported by JSPS KAKENHI, with a Grant Number 21K18086."**

**Please provide an amended statement that declares *all* the funding or sources of support (whether external or internal to your organization) received during this study, as detailed online in our guide for authors at http://journals.plos.org/plosone/s/submit-now. Please also include the statement “There was no additional external funding received for this study.” in your updated Funding Statement.**

**Please expand the acronym “JSPS KAKENHI” (as indicated in your financial disclosure) so that it states the name of your funders in full.**

**Please include your amended Funding Statement within your cover letter. We will change the online submission form on your behalf.**

Response: JSPS stands for Japan Society for the Promotion of Science. KAKENHI is not an abbreviation. We have added the following remarks.

[Added]

There was no additional external funding received for this study.

## 3.

**Thank you for stating the following in the Competing Interests section:**

**"Kagiyama and Daida are affiliated with a department funded by Philips Japan, Asahi KASEI Corporation, Inter Reha Co., Ltd, and Toho Holdings Co., Ltd., based on collaborative research agreements. Other authors have no conflict of interest to declare."**

**Please confirm that this does not alter your adherence to all PLOS ONE policies on sharing data and materials, by including the following statement: "This does not alter our adherence to PLOS ONE policies on sharing data and materials.” (as detailed online in our guide for authors http://journals.plos.org/plosone/s/competing-interests). If there are restrictions on sharing of data and/or materials, please state these. Please note that we cannot proceed with consideration of your article until this information has been declared.**

**Please include your updated Competing Interests statement in your cover letter; we will change the online submission form on your behalf.**

Response: We added the following remarks. No data sharing restrictions exist for our data.

[Added]

Kagiyama and Daida are affiliated with a department funded by Philips Japan, Asahi KASEI Corporation, Inter Reha Co., Ltd, and Toho Holdings Co., Ltd., based on collaborative research agreements. Other authors have no conflict of interest to declare. This does not alter our adherence to PLOS ONE policies on sharing data and materials.

## 4.

**We note that you have indicated that data from this study are available upon request. PLOS only allows data to be available upon request if there are legal or ethical restrictions on sharing data publicly. For information on unacceptable data access restrictions, please see http://journals.plos.org/plosone/s/data-availability#loc-unacceptable-data-access-restrictions. In your revised cover letter, please address the following prompts:**

### **a)**

**If there are ethical or legal restrictions on sharing a de-identified data set, please explain them in detail (e.g., data contain potentially identifying or sensitive patient information) and who has imposed them (e.g., an ethics committee). Please also provide contact information for a data access committee, ethics committee, or other institutional body to which data requests may be sent.**

Response: We revised the statement as below.

[Original]

Data are available from the corresponding author upon an appropriate request.

[Revised]

The data are not publicly open due to privacy concerns. Sharing the present data with researchers/companies outside the author’s research group is not approved by the IRB at Juntendo University, and IRB application and approval upon an appropriate request are necessary before sharing the data (contact information: kgnb_27_hot@yahoo.co.jp and junkanki@juntendo.ac.jp).

# Review Comments to the Author

**Please use the space provided to explain your answers to the questions above. You may also include additional comments for the author, including concerns about dual publication, research ethics, or publication ethics. (Please upload your review as an attachment if it exceeds 20,000 characters)**

## Reviewer #1:

### **Abstract:**

**The CI 95% of the performance indices are missing. Moreover, the PPV, NPV, and Kappa must be added as important performance indices to the abstract and paper.**

Response: Thank you for the valuable suggestion. We calculated the confidence intervals using the Wilson’s method (1), which is recommended by several papers (2, 3), as shown below. We were not able to include all metrics for all examinations (i.e. for full-zone patient-level, 8-zone patient-level, and zone-level) in the abstract, so PPV and Kappa for the secondary outcomes were included only in the manuscript text.

1 E.B. Wilson. Probable inference, the law of succession, and statistical inference. J Am Stat Assoc 1927;22:209–212.

2 A. Agresti and B.A. Coull. Approximate is better than "exact" for interval estimation of binomial proportions. Am Statistician, 1998;52:119-126.

3 L.D. Brown, T.T. Cai and A. Dasgupta Interval estimation for a binomial proportion Statistical Science, 2001;16(2):101-133

[Original in Abstract]

The patient-level accuracy of 12-zone AI-POCUS for detecting CT-validated pneumonia was 94.5%, sensitivity was 92.3%, and specificity was 100%. When simplified with 8-zone scan, the accuracy, sensitivity, and sensitivity were 83.9%, 77.5%, and 100%, respectively. The zone-level accuracy, sensitivity, and specificity of AI-POCUS were 65.3 %, 37.2%, and 97.8 %, respectively.

[Revised]

The 12-zone AI-POCUS for detecting CT-validated pneumonia in the patient-level showed the accuracy of 94.5% (85.1% – 98.1%), sensitivity of 92.3% (79.7% – 97.3%), specificity of 100% (80.6% – 100%), positive predictive value of 95.0% (89.6% - 97.7%), and Kappa of 0.33 (0.27 – 0.40). When simplified with 8-zone scan, the accuracy, sensitivity, and sensitivity were 83.9% (72.2% – 91.3%), 77.5% (62.5% – 87.7%), and 100% (80.6% – 100%), respectively. The zone-level accuracy, sensitivity, and specificity of AI-POCUS were 65.3% (61.4% – 69.1%), 37.2% (32.0% – 42.7%), and 97.8 % (95.2% – 99.0%), respectively.

**The percentage of CT-validated pneumonia must be added to the subject (65) and frame data (577 lung zones).**

Response: We included them in the manuscript.

[Added in Abstract]

The CT-validated pneumonia was seen in 71.4% of patients at total 577 lung zones (53.3%).

**If random sampling and proper sample size estimation are not used, they must be mentioned as important issues that avoid the generalization to the entire population in the abstract and discussion.**

Response: We added the following limitation in the abstract as well as discussion.

[Added in Abstract]

The sample size calculation was not performed given the retrospective all-comer nature of the study.

### **Material and Methods:**

**The flowchart of the experimental design (including the inclusion and exclusion criteria, etc.) must be added to the paper.**

Response: We added the following flow chart as new Figure 1.

[Original in Results]

A total of 56 subjects (41 patients and 15 controls) were enrolled in the study.

[Added in Results]

During the study period, 45 patients with COVID-19 were admitted to the hospital. After excluding 4 patients who did not undergo lung POCUS due to admission on weekends, 41 patients were enrolled in the study. Including 15 control subjects, a total of 56 subjects (41 patients and 15 controls) were included in the study (Figure 1).

### **Statistical analysis:**

**The CI 95% must be added to the entire indices based on the TRIPOD/STARD guideline. The Kappa (agreement rate) and PPV (the probability of having pneumonia if the test result is positive) must be added.**

Response: Throughout the manuscript, we added the 95% confidence intervals for the performance metrics and PPV and Kappa values. The changes in the results are shown below. The additional statistical analysis was explained as shown below.

[Added in the statistical analysis]

Wilson’s method was used to calculate the 95% confidence interval for each metric (1).

1 E.B. Wilson. Probable inference, the law of succession, and statistical inference. J Am Stat Assoc 1927;22:209–212.

[Original (overall patient-level analysis)]

The overall accuracy, sensitivity, and specificity of the AI-POCUS were 94.5%, 92.3%, and 100%, respectively.

[Revised]

The overall accuracy, sensitivity, specificity, PPV, NPT, and Kappa of the AI-POCUS were 94.5% (85.1% – 98.1%), 92.3% (79.7% – 97.3%), 100% (80.6% – 100%), 100% (90.4% – 100%), 84.2% (62.4 – 94.5%), and 0.89 (0.70 – 0.98), respectively.

[Original (patient-level analysis using only 8 zones)]

The accuracy and sensitivity decreased to 83.9% and 77.5%, respectively; however, the specificity remained at 100%.

[Revised]

The accuracy, sensitivity, NPV, and Kappa decreased to 83.9% (72.2% – 91.3%), 77.5% (62.5% – 87.7%), 64.0% (44.5 – 79.8%), and 0.68 (0.43 – 0.85), respectively; however, the specificity and PPV remained at 100% for both (80.6% – 100% and 89.0% – 100%).

[Original (zone-level)]

The zone-level accuracy, sensitivity, and specificity of the AI-POCUS were 65.3%, 37.2%, and 97.8%, respectively.

[Revised]

The zone-level accuracy, sensitivity, specificity, PPV, NPV, and Kappa of the AI-POCUS were 65.3% (61.4% – 69.1%), 37.2% (32.0% – 42.7%), 97.8% (95.2% – 99.0%), 95.5% (89.6% – 97.7%), 57.5% (52.9% – 61.9%), and 0.31 (0.23 – 0.38), respectively.

**The authors mentioned that the AI method's training, validation, and further details are unavailable. A scientific work must provide the required information to repeat the work. For example, it is unclear which validation framework (hold-out, cross-validation, etc.) was used and whether the results are based on the cross-validated confusion matrix. Moreover, the percentage of the training, validation, and test sets must be provided.**

**The AI method must be briefly described in the paper, and providing the citation is insufficient.**

Response: Thank you for letting us clarify this important point. Although we used an AI application in this study, this development process was performed by the company (Philips) and is not publicly open. In this study, we applied this already-developed commercially available software to our population that is completely different from what the company used to develop the model. In this term, this study can be considered as an external or real-world validation of the model, not the development of the model. As a validation study, we believe that the pipeline and study flow of our study are in line with other studies (e.g., references 1-3) and are in a scientific manner.

1. C. Dung-Hung, et al. Crit Care 2022;26(1):215

2. IZ Attia, at al. Int J Cardiol 2021;329:130-135.

3. N, Kagiyama, et al. Echocardiography. 2016;33(5):756-63

### **Results:**

**For the entire indices (plus PPV, NPV, and Kappa), the CI 95% must be provided, showing the precision of the indices.**

Response: We added 95% confidence indices and PPV, NPV and Kappa in results as shown above in the response to the previous comment.

**The objective comparison with the state-of-the-art using proper statistical methods is missing.**

Response: As we explained in the previous response. This is not a study in which we developed a machine-learning model, but we performed an external validation of the machine learning-based application that was already established and fixed. The gold standard was the CT results, and there is no state-of-the-art to be compared with.

**A major issue is how the proposed method performs in practice. The authors must use the sensitivity and specificity of the proposed system and the prevalence of pneumonia in the population (based on the literature) and use the Bayesian theorem to predict the PPV and NPV of the system in the population.**

**Section 2 of the following paper could be easily used by the authors for this estimation:**

**https://www.ncbi.nlm.nih.gov/pmc/articles/PMC8170576/**

Response: We thank the reviewer for this valuable suggestion. The hospitalization rate of COVID-19 in the elderly people in Japan is expected to be 5 – 15% by the Japanese Ministry of Health, Labour and Welfare (https://www.mhlw.go.jp/content/10900000/000964719.pdf). Based on this data, we assumed that the prevalence of pneumonia is 5% in COVID-19 positive patients. Given the following parameters, we calculated the PPV and NPV.

Given the sensitivity of 92.3% and specificity of 100%, and 5% of prior probability;

PPV: 100% (99.9% – 100%)

NPV: 99.6% (99.6% – 99.6%)

We added these results to the manuscript.

[Added in Results]

Finally, we assumed that the prevalence of pneumonia in COVID-19 positive patients is 5% based on the public data from the Japanese Ministry of Health, Labour and Welfare and calculated the estimated PPV and NPV in the real-world. Given the sensitivity of 92.3% and specificity of 100%, and 5% of prior probability, PPV was 100% (99.9% – 100%) and NPV was 99.6% (99.6% – 99.6%).

### **Discussion:**

**And finally, is the proposed diagnosis system clinically reliable?**

Response: Yes. The present study included consecutive patients with the exclusion of less than 10%. We believe that the population represents the real-world COVID-19 population, and the present results are generalizable in patients hospitalized with COVID-19. The good patient-level accuracy is clinically meaningful. However, as we stated in the manuscript as below, there are limitations and further studies are warranted.

[Original in the limitation]

First, this was a single-center study conducted in a university hospital, and included a small number of cases. Larger studies involving various settings are necessary before the current results can be widely implemented. ~~ Next, we studied only hospitalized patients who were considered sicker than those who were cared for at home or at other facilities. The performance of the present application should be tested in less intensively sick patients to uncover it’s applicability for screening purposes.

## Reviewer #2:

**The strength of the paper is well structured, the description of the related work is well done and that results are compared to results of the similar research. At all the work is of certain significance. The paper provides a very powerful message about the "Artificial Intelligence-based Point-of-care Lung Ultrasound for Screening COVID-19 Pneumoniae: Comparison with CT Scans".**

Response: Thank you for your very encouraging comment. We are very happy that you stated that our paper is of certain significance and provides a very powerful message. We’d like to express our appreciation.
